# Dopamine D_2/3_ Binding Potential Modulates Neural Signatures of Working Memory in a Load-Dependent Fashion

**DOI:** 10.1523/JNEUROSCI.1493-18.2018

**Published:** 2019-01-16

**Authors:** Alireza Salami, Douglas D. Garrett, Anders Wåhlin, Anna Rieckmann, Goran Papenberg, Nina Karalija, Lars Jonasson, Micael Andersson, Jan Axelsson, Jarkko Johansson, Katrine Riklund, Martin Lövdén, Ulman Lindenberger, Lars Bäckman, Lars Nyberg

**Affiliations:** ^1^Wallenberg Centre for Molecular Medicine,; ^2^Umeå Center for Functional Brain Imaging,; ^3^Department of Radiation Sciences,; ^4^Department of Integrative Medical Biology, Umeå University, Umeå S-90187, Sweden,; ^5^Aging Research Center, Karolinska Institutet and Stockholm University, S-113 30 Stockholm, Sweden,; ^6^Center for Lifespan Psychology, Max Planck Institute for Human Development, Berlin D-14195, Germany, and; ^7^Max Planck UCL Centre for Computational Psychiatry and Ageing Research, Berlin, Germany, and London D-14195, United Kingdom

**Keywords:** aging, dopamine, fMRI, PET, working memory

## Abstract

Dopamine (DA) modulates corticostriatal connections. Studies in which imaging of the DA system is integrated with functional imaging during cognitive performance have yielded mixed findings. Some work has shown a link between striatal DA (measured by PET) and fMRI activations, whereas others have failed to observe such a relationship. One possible reason for these discrepant findings is differences in task demands, such that a more demanding task with greater prefrontal activations may yield a stronger association with DA. Moreover, a potential DA–BOLD association may be modulated by task performance. We studied 155 (104 normal-performing and 51 low-performing) healthy older adults (43% females) who underwent fMRI scanning while performing a working memory (WM) *n*-back task along with DA D_2/3_ PET assessment using [^11^C]raclopride. Using multivariate partial-least-squares analysis, we observed a significant pattern revealing positive associations of striatal as well as extrastriatal DA D_2/3_ receptors to BOLD response in the thalamo–striatal–cortical circuit, which supports WM functioning. Critically, the DA–BOLD association in normal-performing, but not low-performing, individuals was expressed in a load-dependent fashion, with stronger associations during 3-back than 1-/2-back conditions. Moreover, normal-performing adults expressing upregulated BOLD in response to increasing task demands showed a stronger DA–BOLD association during 3-back, whereas low-performing individuals expressed a stronger association during 2-back conditions. This pattern suggests a nonlinear DA–BOLD performance association, with the strongest link at the maximum capacity level. Together, our results suggest that DA may have a stronger impact on functional brain responses during more demanding cognitive tasks.

**SIGNIFICANCE STATEMENT** Dopamine (DA) is a major neuromodulator in the CNS and plays a key role in several cognitive processes via modulating the blood oxygenation level-dependent (BOLD) signal. Some studies have shown a link between DA and BOLD, whereas others have failed to observe such a relationship. A possible reason for the discrepancy is differences in task demands, such that a more demanding task with greater prefrontal activations may yield a stronger association with DA. We examined the relationship of DA to BOLD response during working memory under three load conditions and found that the DA–BOLD association is expressed in a load-dependent fashion. These findings may help explain the disproportionate impairment evident in more effortful cognitive tasks in normal aging and in those suffering dopamine-dependent neurodegenerative diseases (e.g., Parkinson's disease).

## Introduction

Previous studies indicate that dopamine (DA) is involved in several cognitive functions [e.g., working memory (WM); Bäckman et al., 2011], episodic memory (Nyberg et al., 2016), and reward learning (Jonasson et al., 2014; de Boer et al., 2017) and influences such complex cognitive processes while modulating the blood oxygenation level-dependent (BOLD) signal, suggesting a chain that progresses from neuromodulation through functional activation to cognitive performance.

Studies in which positron emission tomography (PET) imaging of DA release is integrated with BOLD imaging during cognitive performance reveal a positive association between DA and local neural activity in the nucleus accumbens and substantia nigra during reward-related tasks (Knutson and Gibbs, 2007; Buckholtz et al., 2010). The local DA–BOLD association has been extended to more distal brain regions (Nyberg et al., 2009), given abundant dopaminergic innervation to neocortical areas. Relatedly, *in vivo* markers of the DA system have also been shown to modulate functional synchronicity between distal brain regions (Rieckmann et al., 2011, 2012; Cole et al., 2013). However, some studies have failed to observe an association between markers of the DA system and BOLD activity (D'Ardenne et al., 2008; Guitart-Masip et al., 2015). A possible reason for the discrepant findings is differences in task demands, such that a more demanding task requiring greater prefrontal activation (Cabeza and Nyberg, 2000) likely yields a stronger association with DA. Toward this end, a previous study showed a strong positive association between DA D_1_ binding potential (BP) and accuracy in the multisource interference task only during the more demanding interference condition (Karlsson et al., 2009). Similarly, Parkinson's disease (PD; which is characterized by gradual DA depletion) is accompanied by disproportionate impairment in effortful cognitive tasks (Weingartner et al., 1984).

Because of relatively few D_2/3_ receptors outside striatum (Farde et al., 1988; Hall et al., 1994), assessment of extrastriatal D_2/3_ BP using the most common radioligand (raclopride) has not been considered feasible, and previous studies have therefore focused only on striatal D_2/3_. The function of the DA D_2/3_ receptor has been related to different aspects of working memory (Cools and D'Esposito, 2011), and computational models have emphasized the role of striatal DA D_2/3_ in selective updating of WM (Hazy et al., 2006). Similarly, human neuroimaging and animal studies have demonstrated an effect of striatal D_2/3_ on striatal BOLD activation and frontoparietal connectivity during attentional shifting and WM (Clatworthy et al., 2009; van Schouwenburg et al., 2010). However, despite the initial skepticism regarding the specificity of raclopride in measuring extrastriatal D_2/3_ BP, a recent study showed very good test–retest reliability for D_2/3_ BP in extrastriatal regions (Alakurtti et al., 2015). We also showed excellent model fit for D_2/3_ BP using raclopride such that both striatal and extrastriatal regions exhibited factor structures consistent with the known dopaminergic systems (G. Papenberg, L. Jonasson, N. Karalija, Y. Köhncke, M. Andersson, J. Axelsson, K. Riklund, U. Lindenberger, M. Lövdén, L. Nyberg, and L. Bäckman, unpublished observations). The two latter studies suggest that extrastriatal raclopride DA D_2/3_ BP may represent a meaningful signal, and may thus modulate neural responses during WM. Indeed, striatal and extrastriatal DA D_1_ receptor densities have been shown to selectively modulate functional connectivity of WM networks (Roffman et al., 2016), but no such evidence exists for DA D_2/3_ receptors.

Although we recently showed marked individual differences in BOLD modulation as well as in DA D_2/3_ BP as a function of WM performance (Salami et al., 2018), the relationship between the two measures remained unknown. The current study aims to (1) examine whether the potential DA–BOLD association is load dependent and is differentially expressed across different performance subgroups and (2) explore whether striatal and extrastriatal DA D_2/3_ receptors selectively influence the BOLD response in different parts of the brain. Participants were scanned with fMRI while engaging in a numerical *n*-back task, along with DA D_2_ assessment with PET. Latent profile analysis (LPA) on WM accuracy identified two groups. To identify DA–BOLD associations during the three WM load conditions, we applied multivariate spatiotemporal partial-least-squares (PLS) analysis in relation to composite DA D_2/3_ as well as striatal and extrastriatal D_2/3_ measures across two groups.

## Materials and Methods

A detailed description of the recruitment procedure, imaging protocols, and cognitive and lifestyle assessments in the COBRA (Cognition, Brain, and Aging) Study have been published elsewhere (Nevalainen et al., 2015; Nyberg et al., 2016; Lövden et al., 2017; Kaboodvand et al., 2018; Salami et al., 2018; Köhncke et al., 2018). Here, we describe the methods directly relevant to the present study.

### 

#### Participants

The initial sample consisted of 180 older individuals (64–68 years; mean, 66.2; SD, 1.2; 81 women) randomly selected from the population register of Umeå, in northern Sweden. Exclusion criteria included suspected brain pathology, impaired cognitive functioning (Mini Mental State Examination <27), and conditions that could bias the brain measurements (e.g., severe trauma and tumors) and cognitive performance (e.g., severely reduced vision) or preclude imaging (e.g., metal implants). Twenty-eight percent of the sample was working, 18% used nicotine, and 33% took blood pressure medications. The mean education level was 13.3 years (SD, 3.5), body mass index was 26.1 (SD, 3.5), systolic blood pressure was 142 (SD, 17), and diastolic blood pressure was 85 (SD, 10). The sample is representative of the healthy target population in Umeå.

#### Experimental design and statistical analysis

##### In-scanner task.

Performance data (sum of correct responses) were obtained from a numerical *n*-back task. In this task, a sequence of single numbers appeared on the screen. Each number was shown for 1.5 s, with an interstimulus interval of 0.5 s. During every item presentation, participants reported whether the number currently seen on the screen was the same as that shown one, two, or three digits back. A heading that preceded each blocked condition indicated the load level. Participants responded by pressing one of two adjacent buttons with the index or middle finger to reply “yes, it is the same number” or “no, it is not the same number.” A single fMRI run with nine blocks for each condition (1-, 2-, and 3-back) was performed in random order (interblock interval, 22 s), each block consisting of 10 trials that included four matches (requiring a “yes” response) and six nonmatches (requiring a “no” response). The trial sequence was the same for all participants with only two lures (a single 2-back lure within two of the 3-back blocks). The *n*-back blocks were counterbalanced. The mean condition duration was 313, 306, and 296 s, respectively.

##### Behavioral profiling.

**Figure 1.**
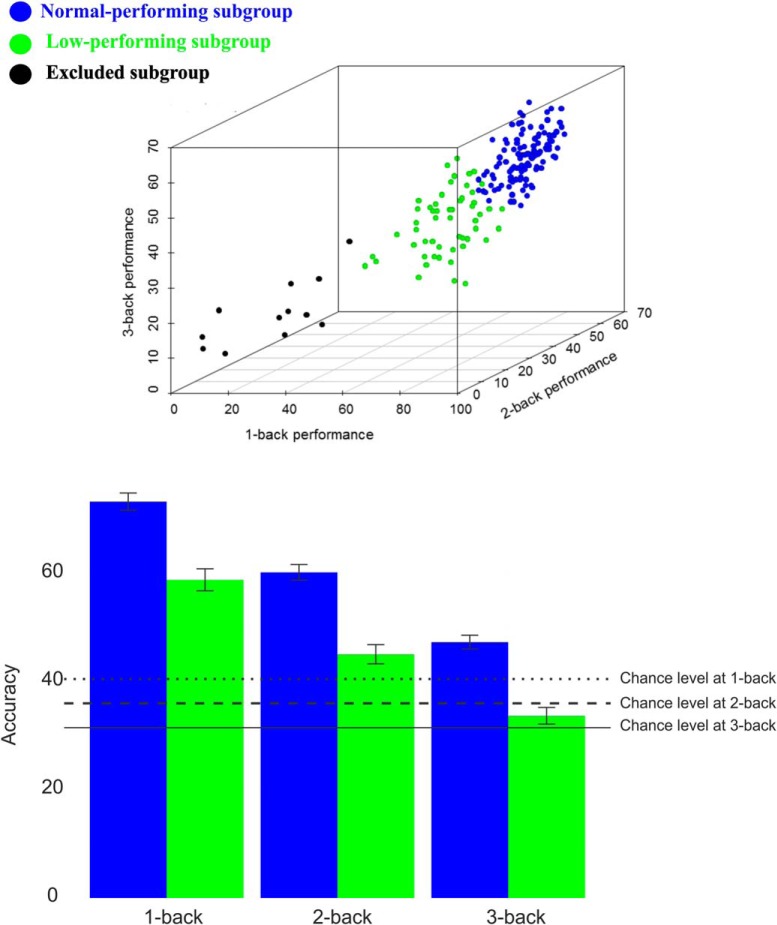
Top, WM subgroups identified by latent-class analysis based on in-scanner *n*-back data for the sum correct during 1-back, 2-back, and 3-back [for more details, see Salami et al. (2018)]. Bottom, The sum of the correct responses across different conditions/groups and their corresponding chance level.

We used LPA, which is a Gaussian mixture-modeling approach for identifying hidden population subgroups (Vermunt and Magidson, 2002). Rather than relying on an arbitrary a priori cutoff, such as the median, LPA is a data-driven method that can operate on multiple indicator variables (1-back, 2-back, and 3-back) to identify latent WM subgroups in the *n*-back data by entering summary scores from each condition as separate variables. Moreover, BOLD–DA performance associations may also be nonlinear and only detectable in certain subgroups. LPA was implemented with Gaussian-mixture modeling. The Bayesian information criterion (BIC) and bootstrap likelihood ratio test were used to compare models with the number of classes varying from one to five. The model with the lowest BIC was selected as the optimal description of latent classes in the data. The analyses were implemented in R's Mclust package (http://cran.r-project.org/web/packages/mclust/index.html). The full details of LPA results have been published recently (Salami et al., 2018). In short, one larger normal subgroup with higher performance (*n* = 113; 63%) and a second smaller subgroup (*n* = 55; 31%) with lower performance were identified (Fig. 1). Interestingly, in a recent study, we found that the low-performing subgroup, identified based on *n*-back performance, displayed lower WM performance across three different off-line tests [i.e., letter updating, numerical 3-back, and spatial updating; see Salami et al. (2018), their Table 2]. No group differences in demographic and vascular factors were found [see Salami et al. (2018), their Table S2]. Note that individuals who performed lower than chance level across all three *n*-back conditions were excluded (*n* = 12, 6%).

#### Image acquisition

Magnetic resonance (MR) imaging was performed with a 3 tesla Discovery MR 750 scanner (General Electric) equipped with a 32-channel phased-array head coil. PET was done with a Discovery 690 PET/CT scanner (General Electric).

##### Structural MR imaging.

A 3D fast-spoiled echo sequence was used for acquiring anatomical T1-weighted images, collected as 176 slices with a thickness of 1 mm (TR, 8.2 ms; flip angle, 12°; field of view, 25 × 25 cm).

##### Functional MR imaging.

BOLD-contrast sensitive scans were acquired using a T2*-weighted single-shot gradient echoplanar imaging sequence. The parameters were as follows: 37 transaxial slices; 3.4 mm thickness; 0.5 mm spacing; TE/TR, 30/2000 ms; flip angle, 80°; field of view, 25 × 25 cm; 96 × 96 acquisition matrix (Y direction phase encoding). At the start, 10 dummy scans were collected. Functional data were acquired during a resting-state condition (6 min), followed by the numerical *n*-back WM task described above.

##### PET image acquisition.

PET was performed during resting-state conditions after an intravenous bolus injection of 250 MBq of [^11^C] raclopride. Preceding the injection, a 5 min low-dose helical computerized tomography scan (20 mA, 120 kV, 0.8 s/revolution) was acquired for attenuation correction. After the bolus injection, a 55 min 18-frame dynamic scan was acquired (9 × 120, 3 × 180, 3 × 260, and 3 × 300 s). Attenuation, scatter, and decay-corrected images (47 slices; field of view, 25 cm, 256 × 256 pixel transaxial images; voxel size, 0.977 × 0.977 × 3.27 mm) were reconstructed with the iterative resolution-recovery VUE Point HD-SharpIR algorithm, using six iterations, 24 subsets, and 3.0 mm postfiltering, yielding full-width at half-maximum (FWHM) of 3.2 mm (Wallstén et al., 2013). Head movements during the imaging session were minimized with an individually fitted thermoplastic mask attached to the bed surface. For 82% of the individuals, PET was performed 2 d after the MR scan (average time difference between MRI and PET was 3 ± 6 d).

#### Image processing

##### PET images.

The following preprocessing steps were performed for each subject in SPM8. The 18-frame PET scans were coregistered to the T1 image using the time frame mean of the PET images as the source. They were then normalized to the MNI space with the subject-specific flow fields (obtained with Diffeomorphic Anatomical Registration Using Exponentiated Lie Algebra (DARTEL)), affine transformed, and smoothed via a Gaussian filter of 8 mm. Normalization parameters were selected so that concentrations in the images were preserved. For determination of D_2/3_ dopamine receptor BP, time-activity curves for each voxel were entered into Logan analyses (Logan et al., 1990), using time-activity curves in the gray-matter parts of cerebellum as reference. Regions of interest were delineated with the FreeSurfer 5.3 segmentation software (Fischl et al., 2002, 2004; Han and Fischl, 2007). Median BP_ND_ data were extracted for all regions of interest based on the subcortical parcellations in Freesurfer and the Desikan–Killiany atlas (Desikan et al., 2006) for extrastriatal regions.

**Figure 2.**
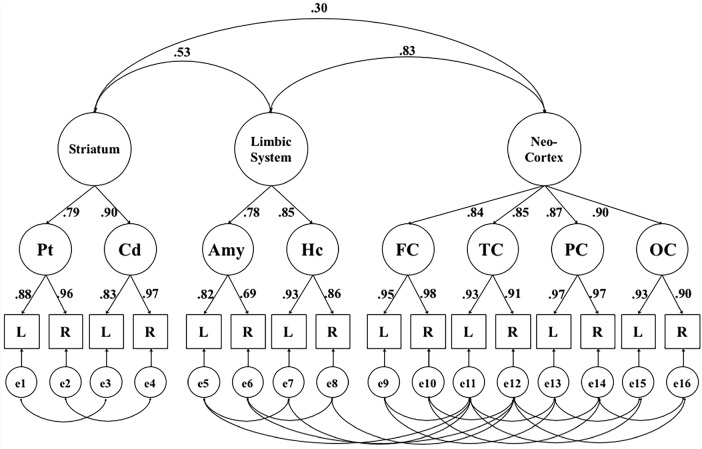
Hierarchical factor model portraying the relationship between [^11^C]raclopride D_2/3_ dopamine receptor BP_ND_ in the striatum, limbic system, and neocortex (Papenberg, Jonasson, Karalija, Köhncke, Andersson, Axelsson, Riklund, Lindenberger, Lövdén, Nyberg, and Bäckman, unpublished observations). Shown are standardized factor loadings and factor correlations for this model. Pt, Putamen; Cd, caudate; Hc, hippocampus; Amy, amygdala; FC, frontal cortex; OC, occipital cortex; TC, temporal cortex; PC, parietal cortex; L, left hemisphere; R, right hemisphere; e, error. Errors represent hemisphere-specific variance and hemisphere-specific measurement error. Model fit parameters are as follows: χ^2^ (77, *n* = 176) = 147.2, *p* < 0.05, Comparative Fit Index (CFI) = 0.97, Root Mean Square Error of Approximation (RMSEA) = 0.074, CI_RMSEA_ (0.056, 092). Values ≤0.08 for RMSEA and ≥0.90 for CFI are considered to indicate a good model fit.

Structural equation modeling was used to model between-person differences in D_2/3_ availability across targeted brain regions belonging to anatomically defined pathways. More specifically, analyses were conducted using AMOS 7.0 (Arbuckle, 2006), modeling latent variances, and covariances. To explore individual differences across anatomical dopaminergic pathways, we estimated a hierarchical model, exploring the factor structure among the striatum, limbic system, and neocortex (Fig. 2; adopted from Papenberg, Jonasson, Karalija, Köhncke, Andersson, Axelsson, Riklund, Lindenberger, Lövdén, Nyberg, and Bäckman, unpublished observations). Following standard notation (Boker et al., 2002), the boxes in Figure 2 indicate the observed variables, the circles represent latent factors, the arrows denote factor loadings, and the double-headed arrows indicate correlations.

One of the advantages of structural equation models is that latent factors are considered free of error, as errors are separately estimated and represented. The latent factors represent the common variance of the variables serving as their indicators, and the error terms represent indicator-specific variance. This increases the statistical power for detecting associations among the constructs of interest, which are represented as latent factors. [^11^C]raclopride BP_ND_ of each region of interest in the left and right hemispheres was used to reflect first-order latent factors, extracting the common variance across hemispheres (Raz et al., 2005). The second-order factors represent the variance that is common to the first-order factors. To define a metric for the factors, one loading on each factor was fixed to 1. Indicators for neocortical latent factors are unit-weighted composite scores based on all available regions in the Desikan–Killiany atlas for frontal, temporal, occipital, and parietal cortex (Desikan et al., 2006). Previously, we have shown that this model representing striatal, limbic, and neocortical second-order latent factors had a good fit (Papenberg, Jonasson, Karalija, Köhncke, Andersson, Axelsson, Riklund, Lindenberger, Lövdén, Nyberg, and Bäckman, unpublished observations). For analyses in the present study, we derived individual values for the limbic, striatal, and neocortical latent factors using regression-based estimation of factor scores. To generate a composite DA measure, a principal component analysis was performed across three factors (i.e., striatum, limbic, and neocortex). This measure reduces model complexity (e.g., similar to the brain score derived from BOLD) in the initial PLS analysis and represents the shared variance across three different factors with a comparable contribution (factor loading for striatal was 0.68, factor loading for limbic was 0.97, and factor loading for neocortical was 0.88).

##### fMRI analyses.

Preprocessing of the fMRI data included slice-timing correction and motion correction by unwarping and realignment to the first image of each volume. The realignment routine calculates three translation parameters (*x*, *y*, and *z*) and three rotation parameters (pitch, roll, and yaw), reflecting the location of each volume compared with the first volume. The fMRI volumes were normalized to a sample-specific template, using DARTEL (Ashburner, 2007), with affine alignment to the MNI standard space and spatial smoothing with an 8 mm FWHM Gaussian kernel.

The preprocessed fMRI data were analyzed with spatiotemporal PLS (McIntosh and Lobaugh, 2004; McIntosh et al., 2004) to assess commonalities and differences in the DA–BOLD association across experimental conditions (1-back, 2-back, and 3-back), type of dopaminergic measure (composite vs striatal, limbic, and neocortical), and performance groups (normal and low performing). PLS determines time-varying distributed patterns of brain activity as a function of experimental variables and DA measures. An identified pattern reflects association changes across all regions of the brain simultaneously rather than tessellations of regions, thus ruling out the need for multiple comparison correction. A detailed description of spatiotemporal PLS analysis for fMRI data has been given in previous reports (Garrett et al., 2010; Salami et al., 2010, 2012, 2013; Grady and Garrett, 2014). In brief, the onset of each stimulus within each block of images (1-back, 2-back, and 3-back) was averaged across blocks for each condition within the two performance subgroups. A cross-block correlation matrix was computed as the correlation between brain activity across experimental conditions and DA D_2/3_ BP across different regions. The correlation matrix was then decomposed using singular-value decomposition (SVD) to identify a set of orthogonal latent variables (LVs) representing linear combinations of the original variables. In the current study, two PLS analyses were performed, and 6 and 18 LVs were estimable for each behavioral PLS analysis either using the composite DA measure (1 DA variable × BOLD measures in the 3 load conditions × 2 performance subgroups) or across three regions (3 DA variables × BOLD measures in the 3 load conditions × 2 performance subgroups), respectively.

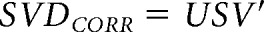
 This decomposition produces a left singular vector of DA D_2/3_ weights (**U**), a right singular vector of BOLD weights (**V**), and a diagonal matrix of singular values (**S**). In other words, this analysis produces orthogonal LVs that optimally represent relationships between DA D_2/3_ and BOLD. Each LV contains a spatial pattern depicting the brain regions in which the activity showed the strongest relation to DA D_2/3_. To obtain a summary measure of each participant's expression of a particular LV pattern, subject-specific “brain scores” were computed by multiplying each voxel's (*i*) weight (*V*) from each LV (*j*) by the BOLD value in that voxel for person (*m*) and summing over all (*n*) brain voxels:

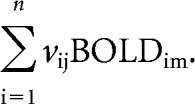
 Together, in a composite-like fashion, a brain score indicates the degree to which each subject contributes to the multivariate spatial pattern captured by a given DA-driven latent variable.

The statistical significance of each LV was assessed using permutation testing, which involved reshuffling the rows of the data matrix and recalculating the LVs of the reshuffled matrix using the same SVD approach. The number of times a permuted singular value exceeds the original singular value yields the probability of significance of the original LV (McIntosh et al., 1996). In the present study, 1000 permutations were performed. In addition, the stability of voxel saliencies contributing to each LV was determined with bootstrap estimation of SEs, using 1000 bootstrap samples (Efron and Tibshirani, 1986). The bootstrap ratio (BSR; the ratio between voxel saliences and the estimated SEs) was computed, and voxels with a BSR >3.29 (akin to a *Z*-score of 3.29, corresponding to *p* = 0.001) were considered reliable. All reliable clusters comprised contiguous voxels, with clusters located at least 10 mm apart from each other. In addition, the upper and lower percentiles of the bootstrap distribution were used to generate 95% confidence intervals (CIs) around the correlation scores to facilitate interpretation (McIntosh and Lobaugh, 2004). For example, a significant difference between correlation scores in different conditions is indicated by non-overlapping CIs. Similarly, brain/correlation scores were deemed unreliable when CIs overlapped with zero. To further explore (within-group) differences in correlation for overlapping CIs, we used an updated version of Steiger's *Z* (http://www.psychmike.com/dependent_correlations.php). Participants with brain scores >3 SDs from the mean were excluded from the analysis, and the PLS value was recalculated. As a result eight participants were excluded from the initial sample with both fMRI and PET (*n* = 163), and thus 155 subjects were included in the final analysis (nomal, *n* = 104; low performing, *n* = 51).

PLS analysis permits the analysis of the relationship between DA and BOLD in one model, without the need of restricting the analyses to specific regions of interest [for a comprehensive report on group differences in DA and BOLD across large-scale networks, see Salami et al. (2018)]. PLS analysis uses all conditions/groups and behavioral measures in an experiment at once and thus offers an additional dimension to data analysis by simultaneously considering indices of both similarities and differences across the experimental variables. If the DA–BOLD association is expressed in similar brain regions, but in different magnitudes across different conditions/subgroups, PLS analysis should reveal a single pattern with quantitative differences across load conditions and groups. Alternatively, if the DA–BOLD association is expressed in different brain regions in a load-dependent manner, we would expect at least two distinct patterns. Similarly, if striatal and extrastriatal DA D_2/3_ selectively modulate the BOLD response, two distinct PLS patterns would be identified. To the extent that DA D_2/3_ modulates neural responses in corticostriatal circuits (which is critical for efficient WM functioning), we predict that a stronger DA–BOLD relationship should be exhibited in individuals with higher WM performance compared with those with lower performance (Salami et al., 2018).

## Results

### Relationship of DA D_2/3_ receptor availability to load-dependent BOLD response during the *n*-back task across performance subgroups

**Figure 3.**
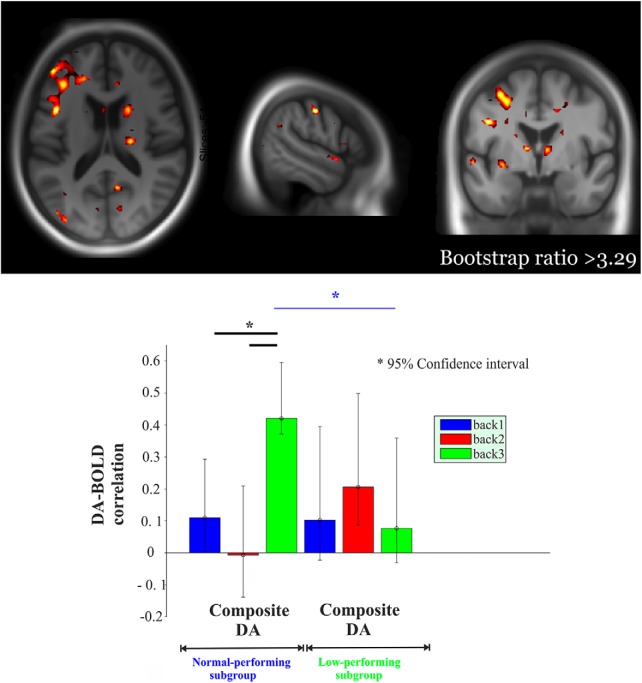
Multivariate relationships of load-dependent BOLD to composite DA D_2_ as the function of performance group. Red regions indicate load-dependent BOLD–DA associations, which are more strongly expressed in the normal-performing group compared with the low-performing group (BSR, >3.29). More specifically, red regions demonstrated a positive and reliable correlation for DA in the normal group during 3-back only. Critically, these regions showed a significantly greater DA–BOLD association during 3-back than during 1-back and 2-back in the normal group. Moreover, this network exhibited a reliable BOLD–DA association during 2-back in the low-performing group. Finally, these red regions showed a stronger DA–BOLD link during 3-back in the normal-performing group compared with the low-performing group. The corresponding correlations across two groups are portrayed in the lower panel. Asterisk represents non-overlapping bars (bars represent 95% confidence interval).

We previously showed less BOLD upregulation in response to increased task demands within the frontoparietal network in the low-performing compared with the normal-performing group [3-back vs 1-back, *t* = 3.48, *p* < 0.001; for whole-brain analysis, see Salami et al. (2018)]. Here, we used PLS analysis to assess whether there was any multivariate spatial pattern of task-related BOLD response dependent on DA D_2/3_ and whether this pattern varied as a function of performance subgroups (normal-performing vs low-performing subgroup). The initial PLS analysis with composite DA D_2/3_ revealed a significant LV (*p* = 0.002; 45.2% of cross-block correlation; Fig. 3). This network demonstrated a positive and reliable correlation for DA D_2/3_ in the normal group during 3-back only (CIs did not cross zero). Critically, a significantly greater positive DA–BOLD link during 3-back than during both 1-back and 2-back was observed in the normal group (nonoverlapping CIs; Fig. 3). In contrast, a positive and reliable BOLD-DA association was observed during 2-back in the low-performing group. No load-dependent modulation of the DA–BOLD association was observed in this group (overlapping CIs), a finding that was also supported by a lack of significance in correlations [Steiger's *Z*_h_ (2-back vs 1-back) = 0.62, *p* = 0.53; Steiger's *Z*_h_ (3-back vs 2-back) = 1.15, *p* = 0.249). Finally, the DA–BOLD association during 3-back (but not 1-/2-back) was strikingly different across the two groups, with a stronger association in the normal-performing group (nonoverlapping CIs; Fig. 3). The network demonstrating a load-dependent DA–BOLD association engaged primarily thalamus, putamen, caudate, parahippocampus, hippocampus, fusiform gyrus, dorsolateral prefrontal, and medial and lateral parietal regions (Fig. 3, top).

**Figure 4.**
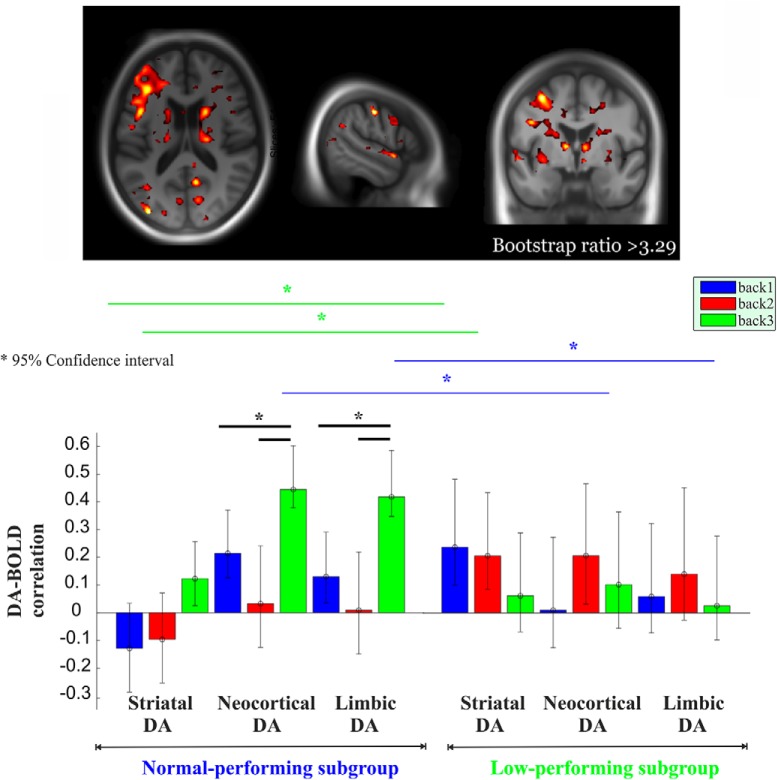
Multivariate relationships of load-dependent BOLD to striatal and extrastriatal DA D_2_ as the function of the performance group. Red regions indicate a load-dependent BOLD–DA association that is more strongly expressed in the normal-performing group compared with the low-performing group (BSR, >3.29). More specifically, the network shown in red demonstrated a significantly greater positive DA–BOLD link during 3-back than during both 1-back and 2-back for extrastriatal regions in the normal group. Similarly, a positive and reliable correlation for DA D_2/3_ in striatum during 3-back was exhibited in the normal group. The size of the DA–BOLD correlation in the red regions for striatum was also marginally greater during 3-back than 1-back and 2-back. In contrast, no significant difference in the DA–BOLD association for striatal and extrastriatal regions was observed in the red regions across different *n*-back conditions in the low-performing group. Despite reliable DA–BOLD associations during 2-back for striatal and extrastriatal regions, no reliable DA–BOLD relationship was observed during 3-back in the low-performing group. Critically, the DA–BOLD association in the red regions during 3-back (but not 1-/2-back) for extrastriatal regions was strikingly different across the two groups, with a stronger association in the normal-performing group. In contrast, the DA–BOLD association during 2-back for striatal regions was greater in the low-performing group. The corresponding correlations across two groups are portrayed in the middle panel (for scatter plots, see Fig. 5). Asterisk represents non-overlapping bars (bars represent 95% confidence interval).

**Figure 5.**
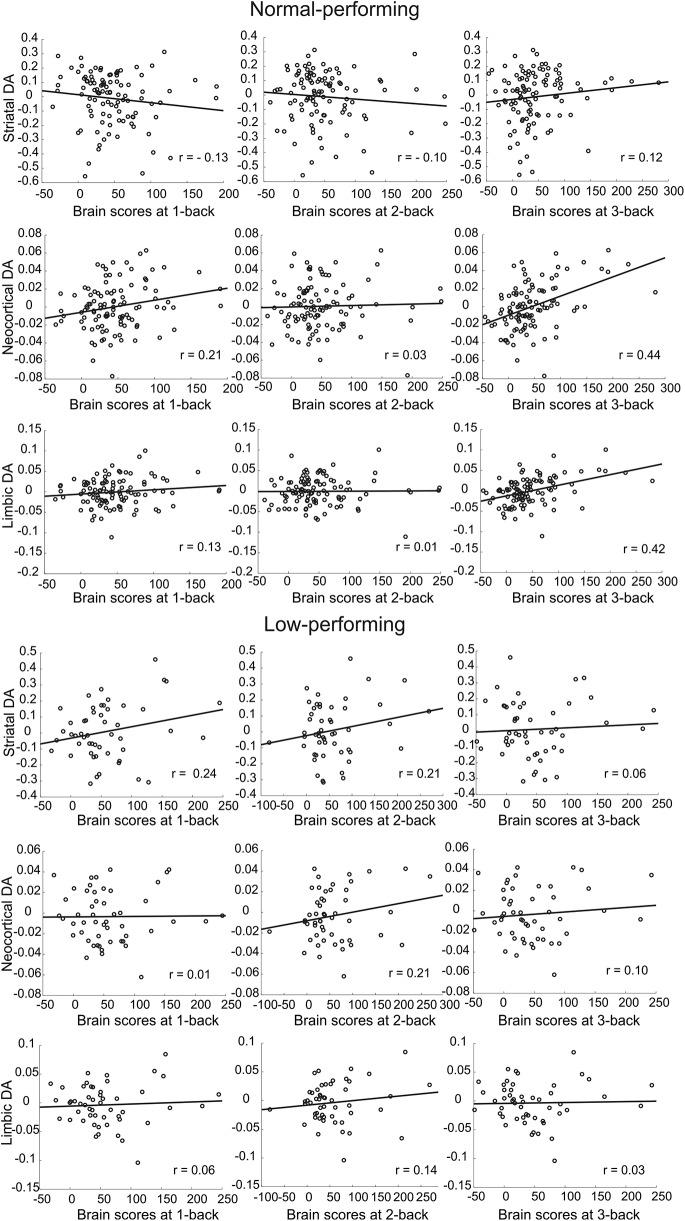
Scatter plot for multivariate relationships of striatal and extrastriatal DA D_2_ to load-dependent BOLD activity across the normal-performing (*n* = 104; top) and low-performing (*n* = 51; bottom) individuals.

In the second PLS analyses using DA D_2/3_ across three different regions, one significant LV was identified (*p* < 0.0001; 43.7% of cross-block correlation; Figs. 4, 5). This network demonstrated a significantly greater positive DA–BOLD link during 3-back than during both 1-back and 2-back for extrastriatal regions in the normal group (Fig. 4, bottom; nonoverlapping CIs). Similarly, a positive and reliable correlation for DA D_2/3_ in striatum during 3-back was observed in the normal group. The degree of DA–BOLD correlation for the striatal region was also marginally greater during 3-back than 1-back and 2-back (despite overlapping CIs, Steiger's test revealed some differences: Steiger's *Z*_h_ (3-back vs 1-back) = 2.1, *p* = 0.035; Steiger's *Z*_h_ (3-back vs 2-back) = 1.91, *p* = 0.056). In contrast, no significant difference in the DA–BOLD association for striatal and extrastriatal regions was observed across different *n*-back conditions in the low-performing group (overlapping CIs; striatal: Steiger's *Z*_h_ (3-back vs 1-back) = 1.06, *p* = 0.29; Steiger's *Z*_h_ (3-back vs 2-back) = 1.25, *p* = 0.21; limbic: Steiger's *Z*_h_ (3-back vs 1-back) = −0.19, *p* = 0.84; Steiger's *Z*_h_ (3-back vs 2-back) = −0.98, *p* = 0.32; neocortex: Steiger's *Z*_h_ (3-back vs 1-back) = 0.54, *p* = 0.58; Steiger's *Z*_h_ (3-back vs 2-back) = −0.91, *p* = 0.36). Despite reliable DA–BOLD associations during 2-back for striatal and extrastriatal regions, no reliable DA–BOLD relationship was observed during 3-back in the low-performing group (Fig. 4, bottom). Critically, the DA–BOLD association during 3-back (but not 1-/2-back) for extrastriatal regions was strikingly different across the two groups, with a stronger association in the normal-performing group (nonoverlapping CIs). In contrast, the DA–BOLD association during 2-back for striatal regions was greater in the low-performing group (nonoverlapping CIs). The network demonstrating a group difference in the load-dependent DA–BOLD link involved thalamus, putamen, caudate, hippocampus, parahippocampus, fusiform gyrus, dorsolateral prefrontal, and medial and lateral parietal regions (Fig. 4, top; Table 1).

**Table 1.** Regions from LV1 showing positive BOLD–DA associations

Region	*x*	*y*	*z*	Bootstrapped ratio	*k* (cluster size)
Left precuneus/left hippocampus	−14	−38	4	5.82	4196
Left thalamus	−10	−4	6	5.25	Subcluster
Left inferior frontal	−44	12	24	5.02	Subcluster
Left superior temporal	−52	6	−8	4.98	Subcluster
Left parahippocampus	−30	−36	−10	4.69	Subcluster
Left caudate	−14	8	10	4.65	Subcluster
Left precentral	−42	−2	30	4.61	Subcluster
Left middle frontal	−38	50	14	4.13	Subcluster
Left superior frontal	−24	60	10	4.12	Subcluster
Left middle frontal	−34	56	2	4.12	Subcluster
Left putamen	−26	8	16	3.94	Subcluster
Left middle frontal	−38	34	30	3.67	Subcluster
Right fusiform	28	−42	−8	5.57	120
Left middle temporal	−50	−68	8	5.47	334
Right calcarine	10	−58	12	5.45	51
Left postcentral	−54	−18	44	5.30	803
Left middle frontal	−32	−2	52	5.11	Subcluster
Left middle occipital	−36	−84	12	5.23	97
Right thalamus	20	−18	14	4.31	Subcluster
Right caudate	22	22	8	3.81	Subcluster
Right parahippocampus	16	−24	−12	4.10	Subcluster
Left middle cingulum	−2	10	44	4.22	53
Right putamen	28	12	−4	4.40	435
Left calcarine	−6	−76	12	4.32	53
Right inferior temporal	52	−56	−12	4.20	73
Right calcarine	12	−76	14	4.17	81
Right anterior cingulum	10	32	16	4.16	62
Right precuneus	8	−42	18	4.06	58
Right superior temporal	58	−14	2	4.02	84
Right superior frontal	26	44	24	3.85	80
Right inferior frontal	46	24	20	3.80	95
Left middle frontal	−32	24	44	3.72	65

Details of the peak voxel of each cluster that resulted from thresholding the brain salience images by a bootstrap ratio of 3.29 (akin to a *z*-score of 3.29, corresponding approximately to *p* = 0.001). Only clusters that included at least 50 voxels are reported.

## Discussion

The primary goal of this study was to examine associations between DA D_2/3_ [^11^C]raclopride PET and BOLD responses during an *n*-back WM task in a large age-homogenous sample of older adults in their mid-60s. Extending previous work in which DA was shown to impact performance particularly in more demanding cognitive tasks, our multimodal imaging approach allowed us to additionally measure a systems-level response in terms of BOLD signal change. Conceivably, this lies between the molecular-level release of the neuromodulator, on the one hand, and cognitive performance, on the other. Using multivariate PLS analysis, we found evidence that both striatal and extrastriatal D_2/3_ receptor density are indicators of the neural responsiveness (i.e., BOLD upregulation) in thalamo–striatal–cortical circuits, which support WM functioning. Our findings extend previous human studies on D_1_ receptor system and striatal synthesis capacity (Landau et al., 2009; Bäckman et al., 2011) by showing that markers of D_2/3_ density are an indicator of the ability to upregulate the BOLD response in spatially distinct cortical regions. Interestingly, we also observed DA–BOLD associations in extrastriatal areas. This finding is in line with a recent study showing that both D_2_ and D_3_ receptor antagonists and agonists modulate blood flow changes in cortical areas (Black et al., 2002; Selvaggi et al., 2018; van den Brink et al., 2018). Indeed, it is well documented that dopaminergic terminals are located close to the cortical microvasculature and *in vivo* administration of DA causes a vasomotor response (Krimer et al., 1998). Our data also align with recent reports that extrastriatal raclopride binding is functionally relevant (Nyberg et al., 2016; Lövdén et al., 2017), despite some remaining uncertainties regarding its specificity. Future research is required to fully elucidate the specificity of extrastriatal raclopride binding more carefully in pharmacological and animal studies. Nevertheless, our study provides early evidence that cortical D_2_/D_3_ densities are critically important for maintaining a responsive frontoparietal WM system. Accurately understanding systems-level responses during cognitive tasks in terms of their molecular basis is important because neuromodulators such as the dopaminergic system are modifiable by drugs or other interventions.

Of critical importance, DA–BOLD associations were expressed in a load-dependent fashion, with stronger associations during 3-back than for 1-/2-back. This pattern was more pronounced in the subgroup with relatively higher WM performance, suggesting that a stronger load-dependent DA–BOLD association is linked to efficient WM functioning. The fact that associations between the task response and marker of the DA system emerge at higher task loads could be linked to the role of DA as a gating mechanism that updates goal representations in prefrontal cortex (Hazy et al., 2006). Demands for updating should be higher during 3-back than 1-/2-back, supported by stronger functional cross talk between striatum and prefrontal cortex during 3-back (Salami et al., 2018), suggesting a greater need for dopaminergic regulation during a more demanding *n*-back condition. At the behavioral level, we recently found that DA D_2/3_ is associated with both working (Lövden et al., 2017) and episodic (Nyberg et al., 2016) memory, but not processing speed, which is presumably a less demanding task. Moreover, past studies of patients with PD showed a relatively greater impairment during a more demanding task (Weingartner et al., 1984). Our findings, in concert with observations from past behavioral studies, suggest that DA has a stronger impact on performance in more demanding cognitive tasks, and we provide novel evidence that this relationship is reflected in a maintained upregulation of spatially distributed cortical association areas engaged during the task.

A previous study provided evidence for a relationship between DA and dorsolateral prefrontal cortex (DLPFC) efficiency (Nyberg et al., 2013) by demonstrating that younger adults and Met carriers of the COMT polymorphism (i.e., high DA) showed maximal DLPFC BOLD response during manipulation of information in WM, whereas older adults and Val carriers (i.e., low DA) exhibited increased DLPFC response during a less demanding maintenance condition. Whereas inferences on DA system integrity were based on differences in age and COMT *val*/*met* allele composition in this previous study, we directly measured DA receptor availability using PET in the current age-homogenous sample. Our analysis revealed that the DA–BOLD association was stronger during 3-back in normal-performing groups but stronger during 2-back in the low-performing individuals. In a previous study, we showed a neural correlate of group by WM capacity constraints (Salami et al., 2018). Moreover, the normal-performing individuals performed 3-back significantly above the chance level, whereas the low-performing group performed the 2-back significantly above the chance level (See Fig. 1). These findings, along with our novel observations (i.e., load × group interaction for BOLD–DA association), indicate that DA–BOLD relationships are more clearly disclosed at the maximum capacity level.

Results from PLS analysis showed no support for selective modulation of striatal and extrastriatal DA D_2/3_. This finding is in contrast to a recent study by Roffman et al. (2016), showing that striatal and extrastriatal DA D_1_ selectively influence functional integrity of different large-scale systems during a WM task. Reasons for this discrepancy may stem from age differences across samples, differences in the kind of BOLD measures [BOLD amplitude in the current vs connectivity in the study by Roffman et al. (2016)], or differences in the type of receptor binding examined and the ligands that are used (DA D_2/3_-sensitive raclopride in the current sample), which play different roles in facilitating WM performance. Although BOLD–DA associations were stronger in extrastriatal than striatal regions, these associations were similarly expressed for both striatal and extrastriatal regions within each group. One reason for the stronger BOLD–DA association in extrastriatal regions might be that the WM network was strongly expressed in cortical rather than subcortical regions (Fig. 4).

In conclusion, our findings provide novel and converging evidence for the key regulatory role of striatal and extrastriatal DA D_2/3_ on functional integrity of the thalamo–striatal–cortical circuit in a load-dependent manner.
